# Correction: “Mouse mammary tumor virus is implicated in severity of colitis and dysbiosis in the IL-10-/- mouse model of inflammatory bowel disease”

**DOI:** 10.1186/s40168-024-01850-9

**Published:** 2024-06-21

**Authors:** Heather Armstrong, Mandana Rahbari, Heekuk Park, David Sharon, Aducio Thiesen, Naomi Hotte, Ning Sun, Hussain Syed, Hiatem Abofayed, Weiwei Wang, Karen Madsen, Eytan Wine, Andrew Mason

**Affiliations:** 1 Center of Excellence for Gastrointestinal Inflammation and Immunity Research, University of Alberta, Edmonton, Canada; 2 Department of Internal Medicine, University of Manitoba, Winnipeg, Canada; 3 Department of Medicine, University of Alberta, Edmonton, Canada; 4 Columbia University, New York, USA; 5 Department of Laboratory Medicine & Pathology, University of Alberta, Edmonton, Canada; 6 Li Ka Shing Institute for Virology, University of Alberta, Edmonton, Canada; 7 Department of Physiology, University of Alberta, Edmonton, Canada; 8 Department of Pediatrics, University of Alberta, Edmonton, Canada; 9 Division of Gastroenterology, University of Alberta, Edmonton, AB T6G 2E1 Canada

**Correction** **:** **Microbiome 11, 39 (2023)**

**https://doi.org/10.1186/s40168-023-01483-4**

In the article titled “**Mouse mammary tumor virus is implicated in severity of colitis and dysbiosis in the IL-10**^**−/−**^** mouse model of inflammatory bowel disease**” [1], the results section subheading “**Characterization of MMTV infection in IL‑10**^**−/−**^** mice”,** paragraph 4 incorrectly assigned the T-cell receptor gene and protein names. The original paragraph is as follows:

“Active MMTV infection in mice can be demonstrated by evaluating superantigen activity and observing differences in the cognate TCR-Vβ subsets that react with the specific vSAG [14]. The IL-10^-/-^ model has a mixed genetic background of several mouse strains including the C57BL/6 and sub strains of 129, each with different and partially characterized endogenous *mtv* loci [30]. For example, the C57BL mouse has three full length endogenous genomes, *mtv-8, mtv-9* and *mtv-17*, and sub strains of 129 mice contain combinations of *mtv-1, mtv-3, mtv-8, mtv-9, mtv-11, mtv-13,* and *mtv-17* [14-16]. Eleven of 12 clones were derived from *mtv-9 sag* encoding the vSAG9 (**Figure 2A**) that preferentially binds and expands TCR-Vβ5, TCR-Vβ11, and TCR-Vβ12 lymphocytes [14]. We then evaluated the TCR-Vβ subset distribution in the spleen and colon (**Supplemental Fig. 2**) and found that TCR-Vβ5 and TCR-Vβ12 were expressed in sufficient quantity for analysis, but TCR-Vβ11 constituted less than 0.5% of the population. Significant differences were observed in the colon that were not observed in the spleen (**Supplemental Fig. 2**). Consistent with vSAG9-induced activity, the percentage of TCR-Vβ12 was significantly increased (17.14 vs. 6.45, q = 0.012), and a trend was observed for increased TCR-Vβ5 (IL-10−/− vs. SvEv, 0.63% vs. 0.21%, q = 0.067) in the IL-10^−/−^ versus the SvEv WT colon (**Fig. **2**B**).”

**Legend Figure 2B:**
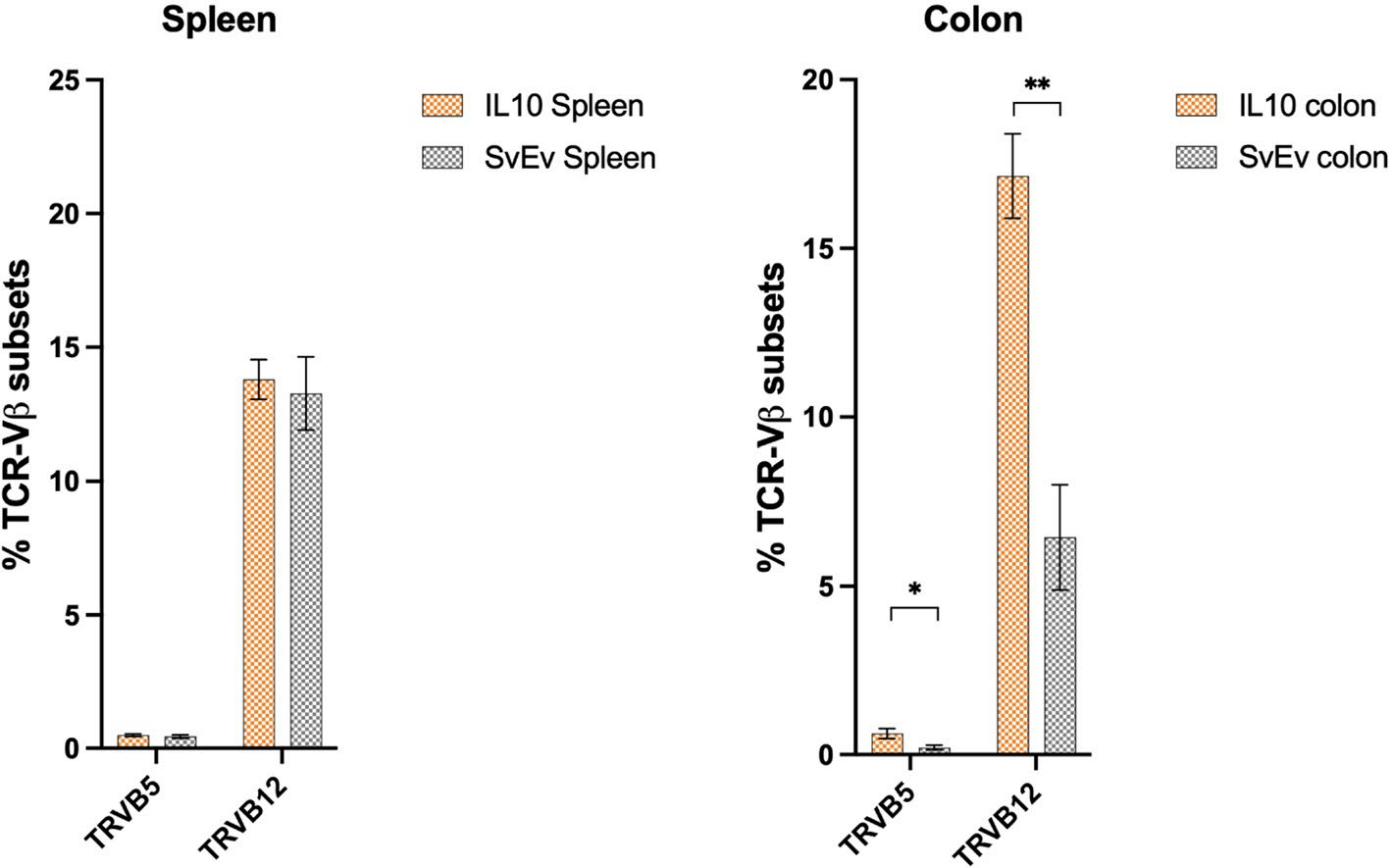
TCR-Vβ5 and TCR-Vβ12 subset distribution of read count assessed by Illumina sequencing in spleen and colon showed no significant differences between IL-10^−/−^ vs. SvEv in the spleen, whereas IL-10^−/−^ colon had more than twofold % increase in TCR-Vβ5 and TCR-Vβ12 (mean ± SEM, **p = 0.006, multiple unpaired t-test, Benjamini, Kreiger, and Yekutieli two-stage setup)

**Supplementary Figure 2**


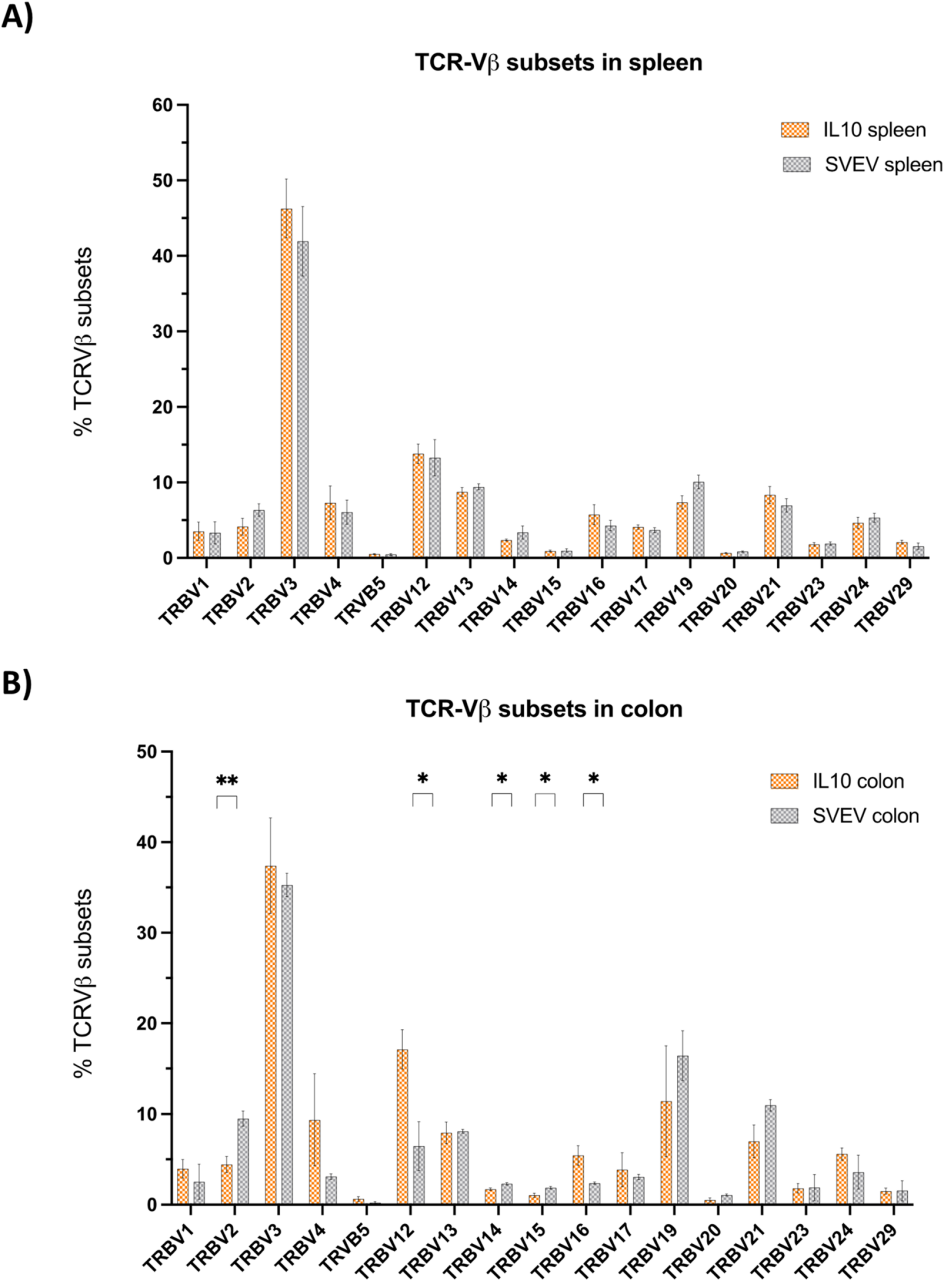


**Supplementary Figure 2 legend**

T cell receptor (TCR)-Vβ subset distribution in spleen and colon of IL-10-/- vs. SvEv mice assessed by Illumina sequencing. (A) No significant differences were observed between IL-10-/- vs SvEv in the spleen. (B) the IL-10-/- colon had increased TCR-Vβ12 and TCR-Vβ16 subsets with diminished TCR-Vβ2, TCR-Vβ14 and TCR-Vβ15 expression. [Mean ± SEM, TCR-Vβ subsets with percent less < 0.5% removed from the analyses. * *p* < 0.01, ** *p* = 0.002, Multiple unpaired t-test, Benjamini, Kreiger, and Yekutieli two stage set up, q value < 0.1].

The correct nomenclature is incorporated into the new paragraph and shown in the revised figure Fig. 2B:

“Active MMTV infection in mice can be demonstrated by evaluating superantigen activity and observing differences in the cognate TCR-Vβ subsets that react with the specific vSAG [14]. The IL-10^-/-^ model has a mixed genetic background of several mouse strains including the C57BL/6 and sub strains of 129, each with different and partially characterized endogenous *mtv* loci [30]. For example, the C57BL mouse has three full length endogenous genomes, *mtv-8, mtv-9* and *mtv-17*, and sub strains of 129 mice contain combinations of *mtv-1, mtv-3, mtv-8, mtv-9, mtv-11, mtv-13,* and *mtv-17* [14-16]. Eleven of 12 clones were derived from *mtv-9 sag* encoding the vSAG9 (**Figure 2A**) that preferentially binds and predominantly expands lymphocytes subsets expressing TCR-Vβ5 and TCR-Vβ11 [14]. We then evaluated the *TRVB* gene distribution in the SvEv WT and IL-10^-/-^ mice in the spleen and colon. We found that the corresponding *TRVB* genes were differentially expressed in sufficient quantity for analysis with significant differences in the colon that were not observed in the spleen (**Supplemental Fig. 2**). Consistent with vSAG9-induced superantigen stimulation, the percentage of *TRVB12* encoding TCR-Vβ5 (17.14 vs. 6.45, p < 0.01) and *TRVB16* encoding TCR-Vβ11 (5.43 vs. 2.37, p < 0.01) were significantly increased in the colon of IL-10^−/−^ vs. SvEv mice.”

**Revised Figure 2B:**
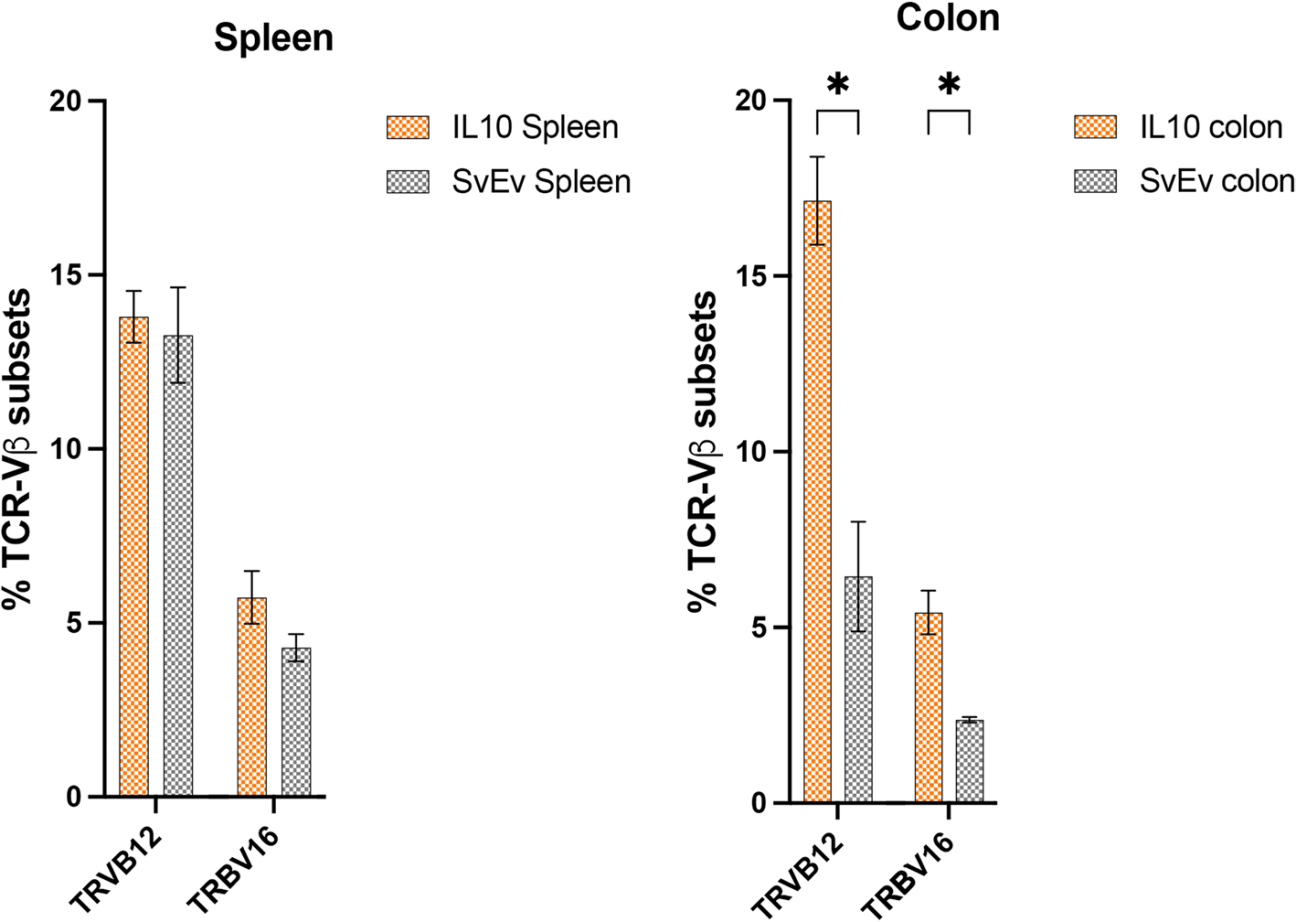
Distribution of *TRVB12* and *TRVB16* gene read counts assessed by Illumina sequencing in spleen and colon. No significant differences were observed in TRBV gene expression between IL-10^−/−^ vs. SvEv in the spleen, whereas in the colon, the IL-10^−/−^ mice demonstrated more than twofold % increase in *TRVB12* and *TRVB16* genes encoding TCR-Vβ5 and TCR-Vβ11, respectively (*p < 0.01, multiple unpaired t-test, Benjamini, Kreiger, and Yekutieli two-stage setup)
