# Supplementary figures and images for: Mouse mammary tumor virus is implicated in severity of colitis and dysbiosis in the IL-10−/− mouse model of inflammatory bowel disease
Source: Microbiome. 2023 Mar 3;11:39. doi: 10.1186/s40168-023-01483-4 (PMC9983191; doi:10.1186/s40168-023-01483-4)

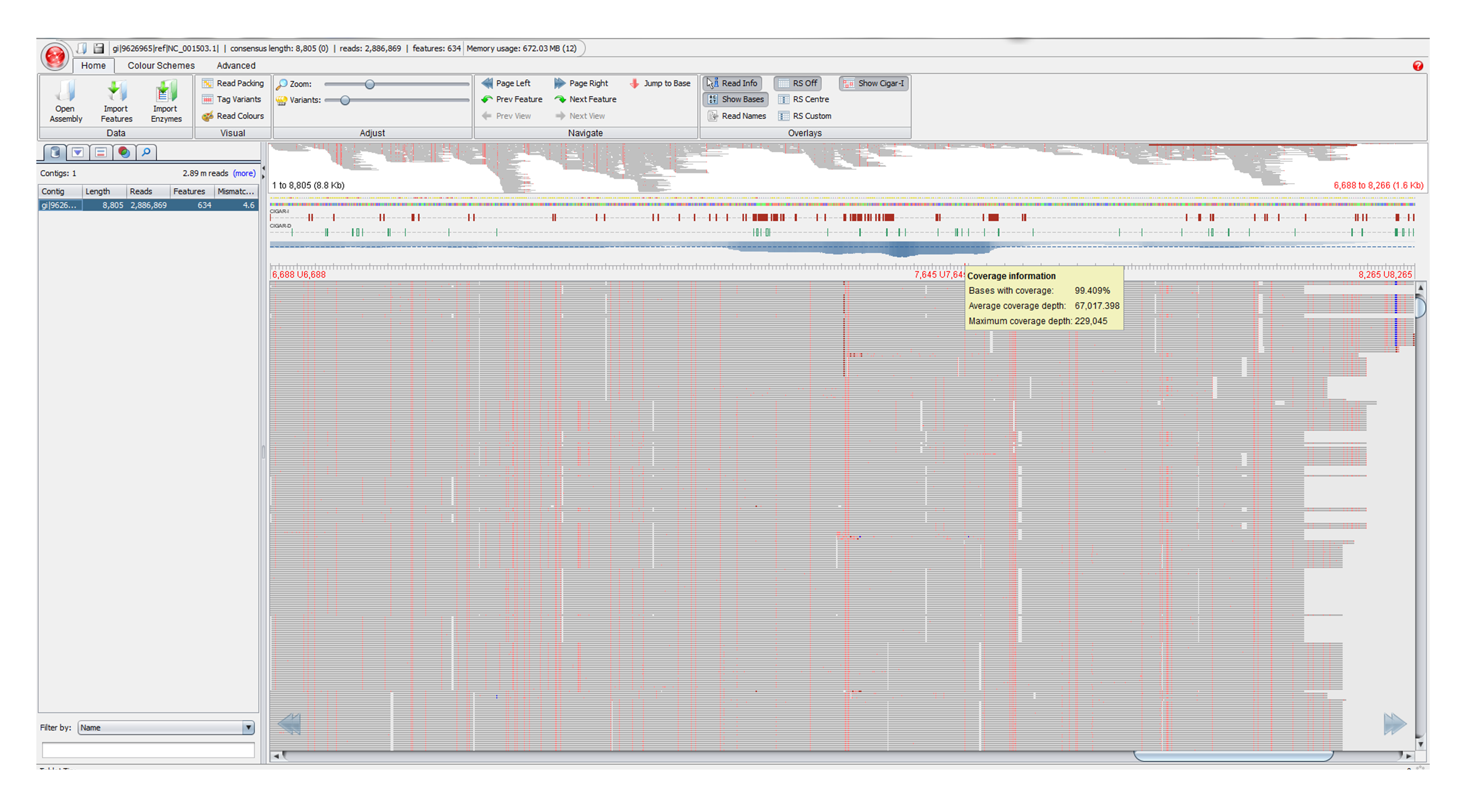

Supplement: Supplementary file 3 — Additional file 2: Supplementary Fig. 1. Alignment of Illumina reads from IL-10-/- lactoserum library to MMTV genome showing a screen capture with alignment of 2,886,869 million Illumina reads along MMTV reference genome with variance in sequence marked in red, to provide 99.40% coverage of the MMTV genome and an average read depth of 67,017-fold. [file 40168_2023_1483_MOESM2_ESM.tif]

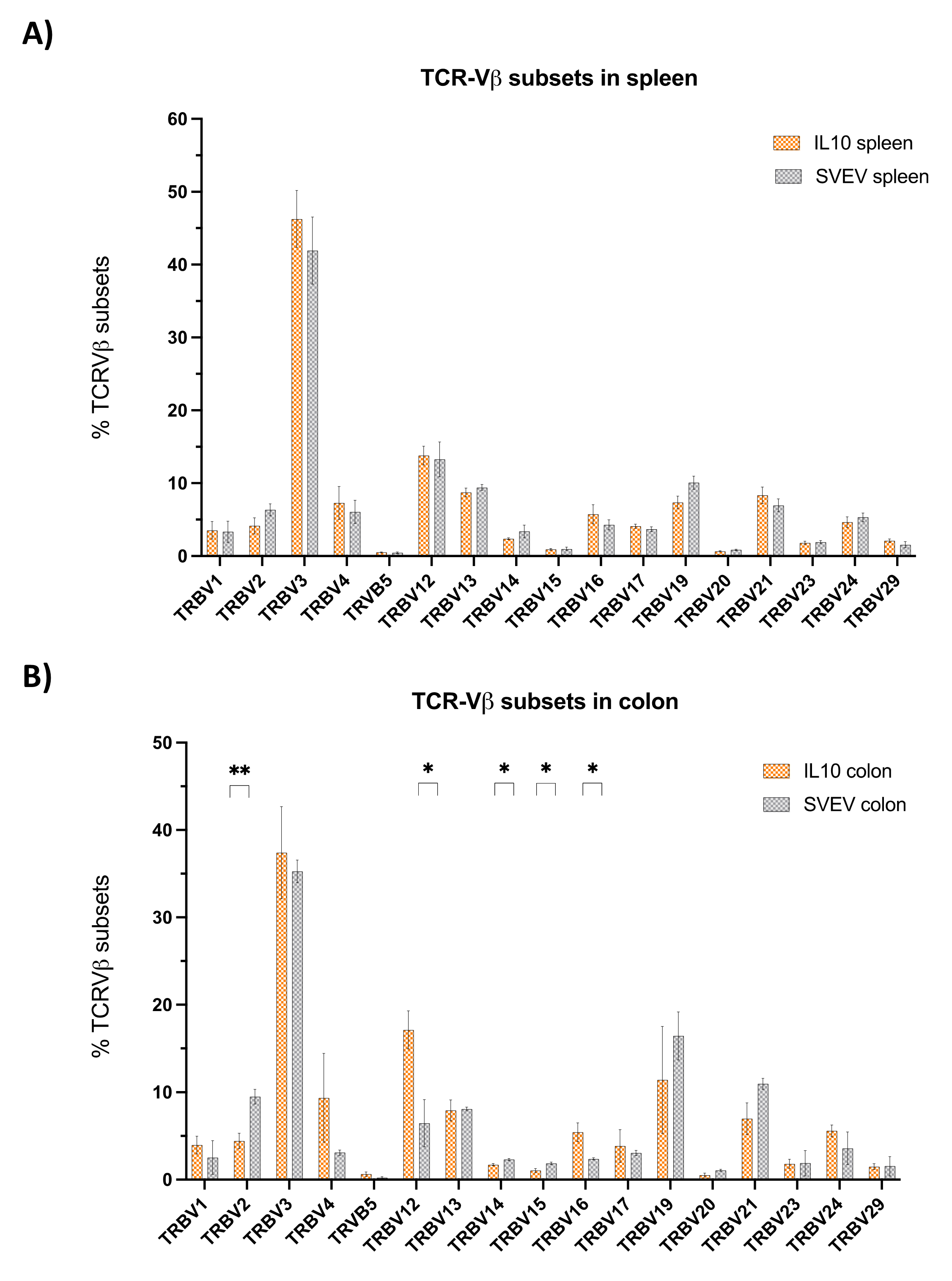

Supplement: Supplementary file 4 — Additional file 3: Supplementary Fig. 2. T cell receptor (TCR)-Vβ subset distribution in spleen and colon of IL-10-/- vs. SvEv mice assessed by Illumina sequencing. (A) No significant differences were observed between IL-10-/- vs SvEv in the spleen. (B) the IL-10-/- colon had increased TCR-Vβ12 and TCR-Vβ16 subsets with diminished TCR-Vβ2, TCR-Vβ14 and TCR-Vβ15 expression. [Mean±SEM, TCR-Vβ subsets with percent less < 0.5% removed from the analyses. * p<0.01, ** p=0.002, Multiple unpaired t-test, Benjamini, Kreiger, and Yekutieli two stage set up, q value < 0.1]. [file 40168_2023_1483_MOESM3_ESM.tif]

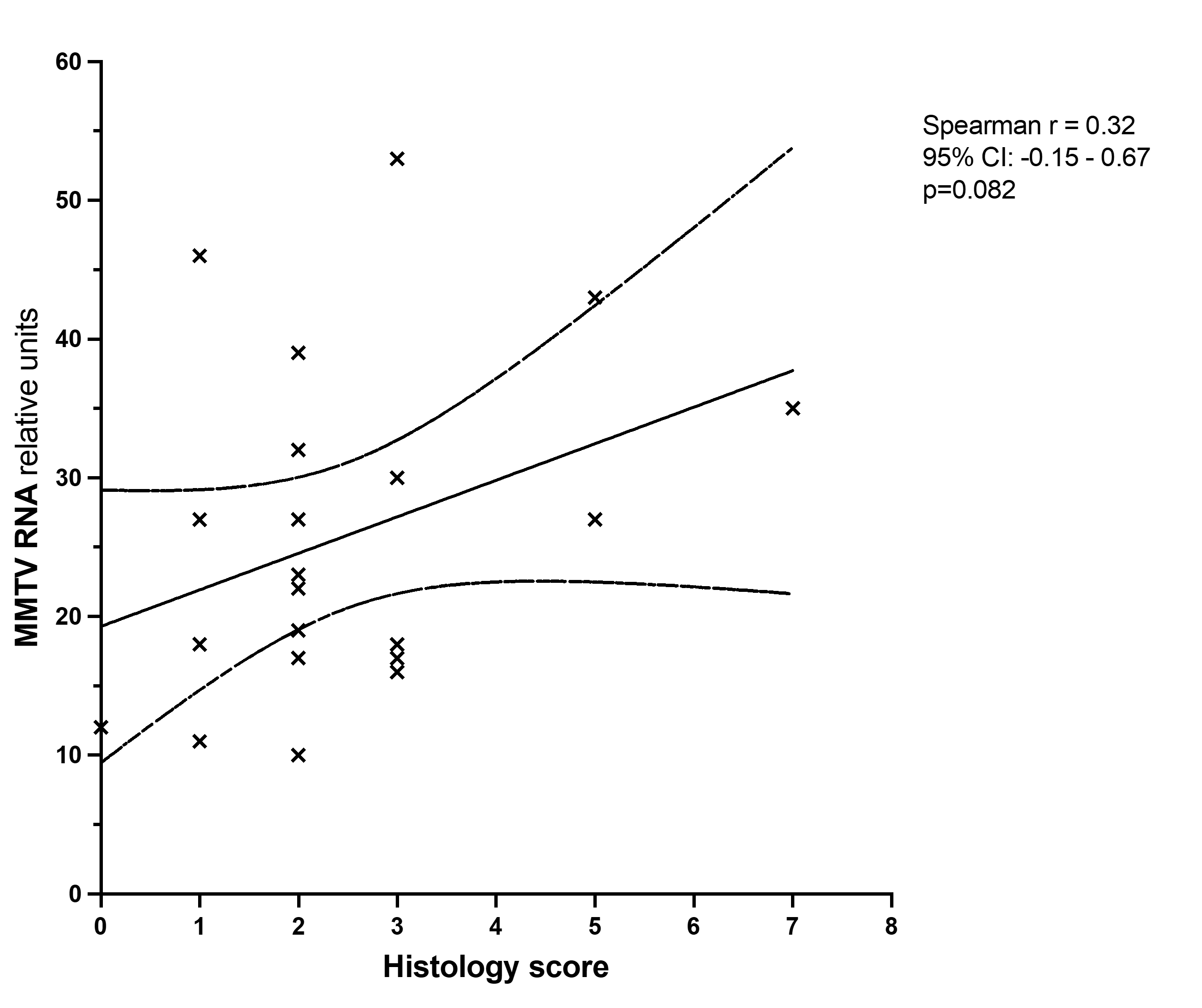

Supplement: Supplementary file 5 — Additional file 4: Supplementary Fig. 3. Correlation of colon MMTV RNA levels and histology score [Spearman, p=0.082]. [file 40168_2023_1483_MOESM4_ESM.tif]

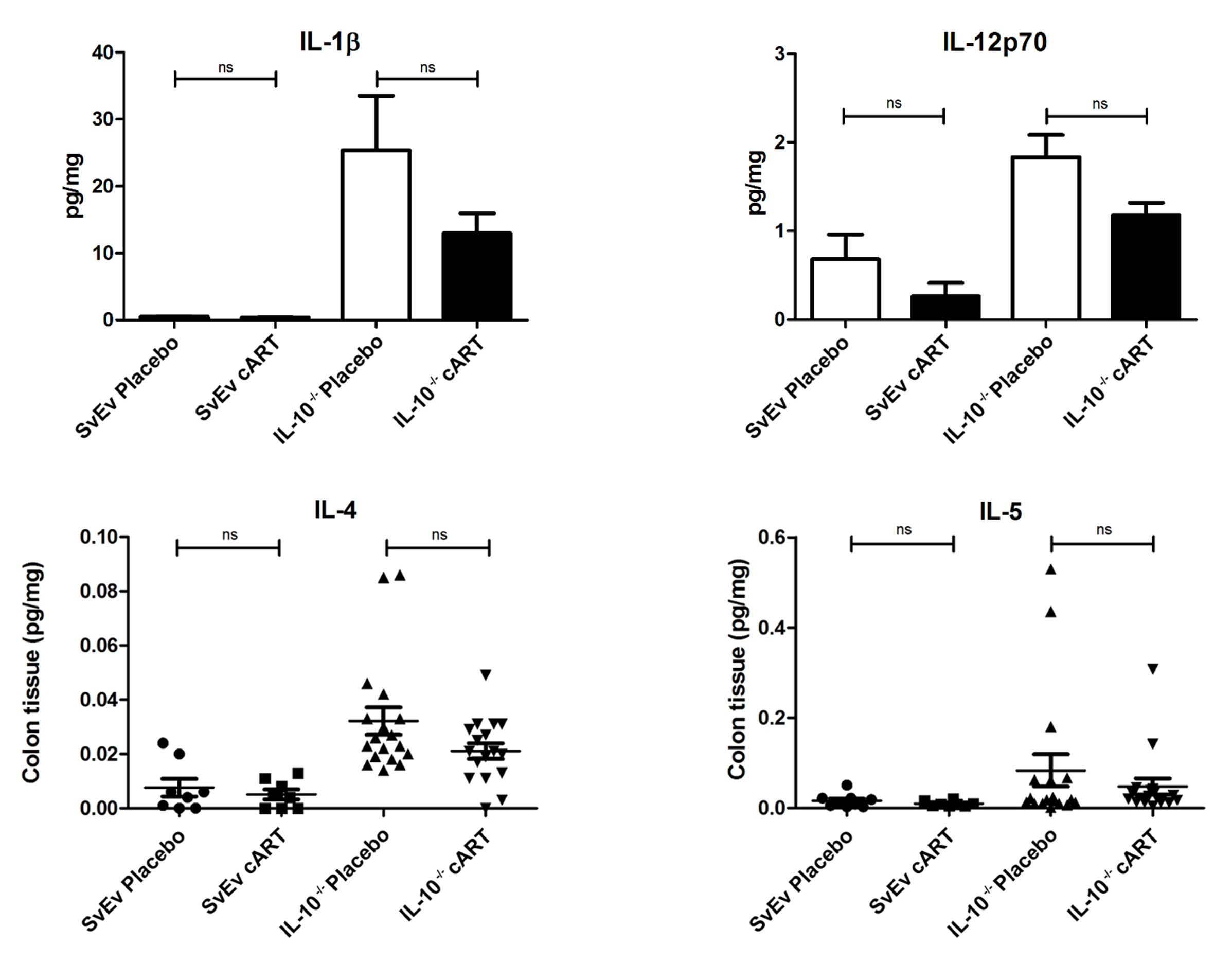

Supplement: Supplementary file 6 — Additional file 5: Supplementary Fig. 4. Pro-inflammatory cytokines in colonic extracts showing that levels of IL-1b, IL-12p70, IL-4, and IL-5 were not significantly altered with cART in the IL-10-/- mice [Mean±SEM]. [file 40168_2023_1483_MOESM5_ESM.tif]
